# A Systematic Review of Empirical Studies on Situation Awareness: Perspectives From the Interaction Among Humans, Machines, and the Task Environment

**DOI:** 10.1002/pchj.70027

**Published:** 2025-07-06

**Authors:** Rong Fang, Qian Zhou, Chen Zhou, Shifang Yuan, Kexin Wang, Qi Li, Yu Zhang, Jie Li

**Affiliations:** ^1^ College of Education Hangzhou Normal University Hangzhou China; ^2^ School of Psychology Beijing Sport University Beijing China; ^3^ School of Sport China University of Mining and Technology Xuzhou China; ^4^ Center for Cognition and Brain Disorders, School of Clinical Medicine Hangzhou Normal University Hangzhou China; ^5^ Department of Psychology and Speech‐Language Pathology University of Turku Turku Finland

**Keywords:** ergonomics, human factors/ergonomics, human‐computer interface, situation awareness

## Abstract

Situational awareness (SA) refers to “knowing what is going on” in a situation. As an essential concept originating in aviation literature approximately 40 years ago, SA has demonstrated significant potential and has since been extended across various fields, leading to a growing body of research. With its expanding application in diverse fields, SA literature has become increasingly fragmented. This study systematically reviews previous empirical studies to provide a structured categorization and comprehensive analysis of SA applications, contributing to the advancement of SA research. Our search identified 2860 empirical studies on SA published between 1975 and 2024, spanning 11 major fields, including aviation, driving, power systems, traffic and transportation, health care and medicine, emergency management, military, training, sport, system autonomy, and network information and communication. We examined the specific characteristics of SA in these fields and, by integrating the characteristics with human factors/ergonomics principles, developed a comprehensive framework. Based on this framework, we categorized the SA research into three groups: the ergonomics‐centered group (e.g., aviation), the human‐centered group (e.g., sports), and the machine‐centered group (e.g., system autonomy). Our findings have the potential to foster collaboration among researchers across diverse fields, facilitating the expansion and integration of SA research through cross‐referencing theories, models, and methodologies.

## 1 Introduction

Situational awareness (SA) refers to “knowing what is going on” in a situation (Endsley 1995). From an information processing perspective, the general definition of SA is “the perception of the elements in the environment within a volume of time and space, the comprehension of their meaning, and the projection of their status in the near future” (Endsley 1995). Additionally, it is also an “adaptive, externally directed consciousness” (Smith and Hancock 1995) from an ecological approach underpinned by the perception–action cycle. SA can be employed by individuals—whether human or machine—as well as by teams and systems composed of multiple humans, machines, or a combination of both, in complex situations (Mansikka et al. 2021; You et al. 2023).

Over the past four decades, SA literature has rapidly grown concerning human factors and cognitive psychology (Endsley 2015; Wickens 2015). As of March 2024, the Web of Science (WOS) database was searched using “situation awareness” as the keyword, yielding 6940 relevant literature articles. These data highlight its substantial attention over the past four decades. While SA originated from ergonomics research in aviation (Endsley 1995), with subsequent development years, it has gradually entered the mainstream lexicon (Stanton, Roberts, et al. 2017; Stanton, Salmon, et al. 2017) and has been explored in various fields, including military (Huttunen et al. 2011; Lee et al. 2023), health care and medicine (Brady and Goldenhar 2014; Walshe et al. 2021), system autonomy (Endsley 2017; McNeese et al. 2018), sports (Bourbousson et al. 2011; Macquet and Stanton 2014), among others. In these studies, the SAs of various agents, including pilots, medical practitioners, athletes, and even autonomous systems, have been extensively investigated.

However, with an increase in its application across diverse fields, the extent of SA application has become increasingly unclear (Cooper et al. 2012; Endsley 2015), lacking a comprehensive overview. Endsley (2015) delineated the expansion of SA from aviation to air traffic control, military operations, transportation, power systems, law enforcement, emergency management, healthcare, space exploration, education, mining, and oil and gas operations (Endsley 2015). Similarly, Salmon et al. (2006) argued that SA has extensive utility in command, control, communication, computers, and intelligence (C4i) systems. However, Endsley and Stanton provided descriptive accounts of SA applications across various fields without conducting a systematic review. Moreover, its application has recently transcended traditional domains and has gradually extended to new areas, such as system autonomy and network information. This trend has recently underscored the progressive expansion of SA into new fields, highlighting the need to investigate this expansion.

Furthermore, the association among studies in various fields of application lacks clarity. Although these utilize the SA concept, significant differences are observed among the application models, theories, and methods (Nguyen et al. 2015; Singh et al. 2019; Endsley 2017). For instance, in aviation, SA is pivotal to pilot training, facilitating flight, aircraft control, and maintenance. The primary measurement methods for SA include freeze‐probe recall, real‐time probe, and post‐trial self‐rating techniques (Nguyen et al. 2015). Concerning the medical domain, recognized as critical to medical personnel's Non‐Technical Skills (NTS), SA affects their technical abilities and clinical outcomes arising from direct interactions with the medical environment (Ghaderi et al. 2023). The primary measurement method used was a quantitative SA analysis. Technological advances have enabled intelligent agents (IAs), such as software or embodied robots, to achieve system autonomy (Schaefer et al. 2017). In this field, IAs can possess their own SA and contribute to its overall system through communication protocol development (Liu et al. 2024; Kim et al. 2023). In system autonomy, a significant shift from other fields is the change in the human role from the machine controller to the supervisor or collaborator. Autonomy involves distinct decision‐making processes, contexts, and communication methods (Ezenyilimba et al. 2023). Therefore, an in‐depth understanding of the developmental history of these fields remains fragmented. Their future directions lack clarity owing to disjointed development trajectories and lacking cohesive overall direction.

To bridge this literature gap, this study systematically reviews SA empirical literature to foster interconnections across various disciplines, facilitating field expansion and integration. First, we searched all empirical studies utilizing the keyword “situation awareness” across the four major databases. Subsequently, we categorized these studies into distinct fields according to keywords, research objectives, and SA agents to ascertain the quantity and specific scope of the prevalent mainstream SA application domains. Furthermore, this review compared the specific application characteristics of each domain from the perspectives of humans, machines, and environments to elucidate their associations and distinctions. Specifically, by integrating the specific application characteristics of each field with classical human factors/ergonomics (HFE) principles, this study synthesized a comprehensive clustering framework categorizing all the fields into three groups. Additionally, this review examines the historical evolution of SA research in these fields and systematically explores future research directions.

## 2 Review Process and Literature Search Method

**FIGURE 1:**
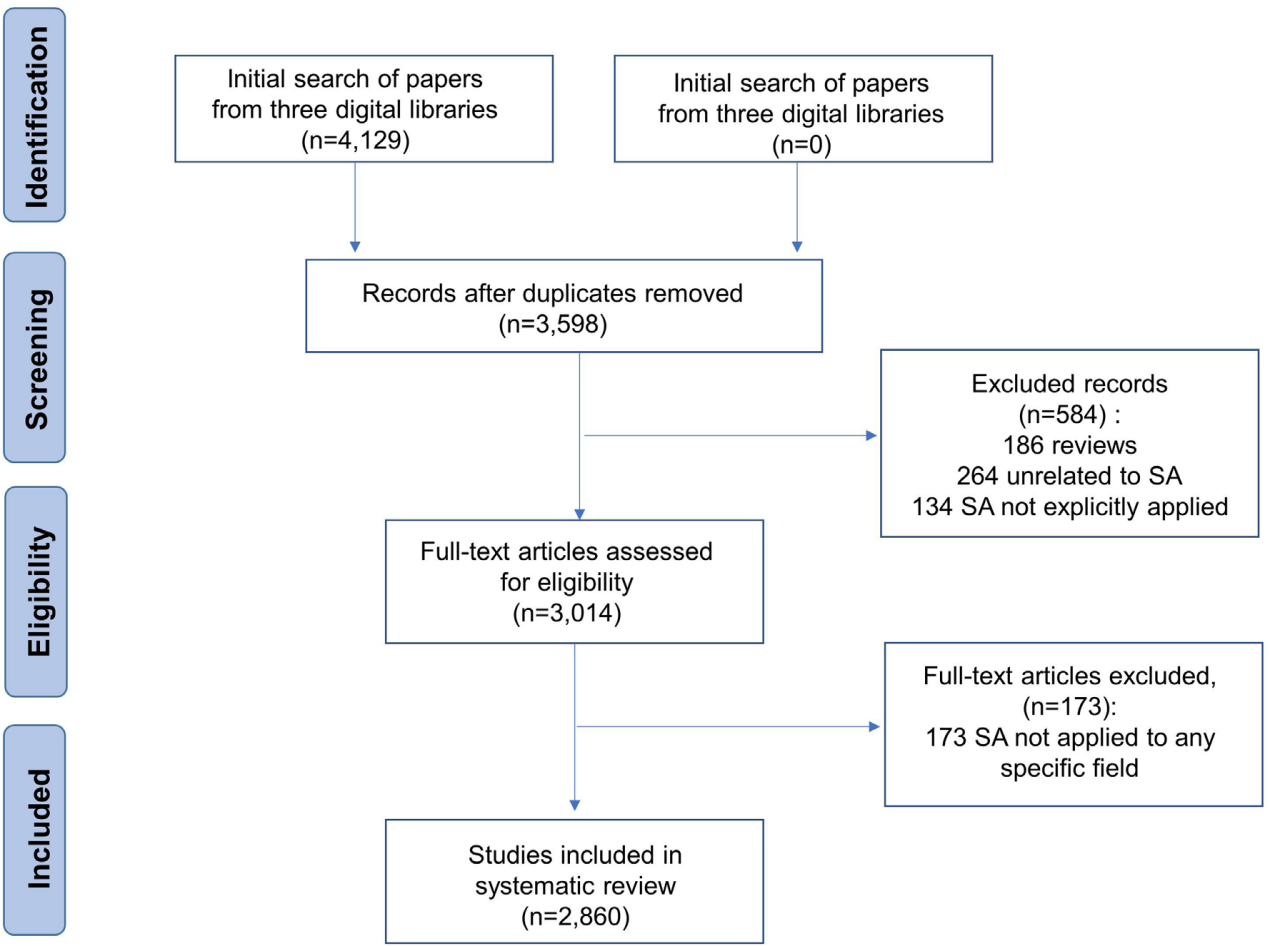
PRISMA flow diagram of the review process: Number of records identified, inclusions and exclusions, and reasons for exclusions.

Following the preferred reporting items for systematic review and meta‐analysis (PRISMA) method (Shamseer et al. 2015), this systematic study aimed to obtain an overview of the SA application status. The overall review process, from defining the review scope to identifying the final selection of articles for analysis, is illustrated in Figure 1. The article review process comprised six steps, as discussed below.

### 2.1 Step 1: Definition of the Review Scope and Keywords

This study followed the procedures suggested by Webster and Watson (2002) by selecting keyword search strategies for relevant digital libraries. The libraries relevant to our search were Web of Science, ProQuest, Scopus, and EBSCO. Other databases, such as Science Direct, IEEE, and Taylor and Francis, were excluded since most of the articles they contained were expected to be included in the selected databases. To ensure an extensive search of all empirical literature applied to SA, we placed our search keyword as “situation awareness.”

Other related SA terms used in other studies include “situation understanding” (Chen et al. 2019; Liao et al. 2017), “situation recognition” (Junger et al. 2022; Li et al. 2017), and “situation prediction” (Hu et al. 2019; Leau and Manickam 2015). Conceptually, these terms each reflect one specific aspect of information processes in complex situations, whereas the SA concept is comprehensive and encompasses the aspects elucidated by these related terms. Regarding quantity, the number of articles employing these terms is relatively limited, and the delineation of domains lacks clarity. Therefore, this review explicitly focused on the term “situation awareness” rather than utilizing the aforementioned related terms.

### 2.2 Step 2: Initial Search in Four Digital Libraries

For our database search, we defined the following search string: “situation awareness” AND NOT review OR meta‐analysis OR “systematic review.” The search results included peer‐reviewed scientific journal articles written in English and published between 1975 and 2024 (up to April 1st). The aggregated results of the initial search included 4129 articles.

### 2.3 Step 3: Removing Duplicate Documents

We first used EndnoteX9 to reduce redundant results by automatically removing duplicates and subsequently, cross‐checked the articles in the database by sorting them by title in Microsoft Excel. After implementing both processes, the number of remaining articles was 3598.

### 2.4 Step 4: Filtering Following the Exclusion and Inclusion Criteria With Title and Abstract

Six authors, divided into three groups, read through the 3598 titles and abstracts obtained, and the articles were screened for exclusion following three criteria: review articles, articles not directly related to SA, and articles wherein the SA application was unclear. To improve the reliability of judgments, at least two authors read each abstract, and in case of disagreement, a third author was consulted until a consensus was reached. Hence, 3014 articles were selected.

### 2.5 Step 5: Establishing Initial Fields and Division Criteria

Subsequently, we established the initial SA application fields and their criteria. Firstly, according to Endsley's classification of SA application fields and Salmon's C4i systems, we set 14 initial SA application fields: aviation, driving, military, traffic control, transportation, power systems, emergency management, health care, education, training, sports, oil mining, system autonomy, and network information security (Endsley 2015; Salmon et al. 2006). Later, we read several representative articles in each field and proposed that a key criterion for SA research in a specific field is that its agent is a typical operator in the field. For example, when SA agents are pilots, runway controllers, or tower controllers, it may be an SA study in the aviation field. Subsequently, we identified an initial set of articles (approximately 30) in each field by searching the field names in the 3014 articles. For example, we selected 30 articles with “aviation” in the titles or keywords as the initial aviation set. By reading the initial sets of papers in the field, we determined two criteria for classifying an article into a field: the agent of SA is the typical operator in this field, and the keywords of the article are related to the field. The SA agents and keywords used in this field are listed in Table 1.

**TABLE 1:** Keywords and agents of fields.

Field	Keywords	Agents
Aviation	Flight, visual flight, cockpit, aerial vehicle, flight deck, automatic pilots, flight deck display, aviation flight displays	Pilots, runway controllers, tower controllers
Driving	Driving, risky driving, contingency, accidents, driving simulator, platooning, automated driving, cruise control, driver distraction, vehicles, adaptive cruise control	Drivers, driver assistance systems
Power system	Nuclear, power, power plant, control rooms, smart grid, power flow, renewable energy, electric power systems, power distribution system	Operator, operations team
T	Traffic, transport, city traffic, maritime safety, traffic engineering, traffic controllers, railway, traffic accident detection, traffic accident, maritime industry, intelligent transportation system, flow prediction, road traffic control, social transportation	Operator, traffic controllers, crew, accident signaling system, control centers
Military	Military, military operations, military science, military personnel, command agent, military vehicle detection response, military command, internet of battlefield things, optimal attack strategy, frontline commanders	Cadets, warfighters, soldiers, infantry squads, incident commanders, higher command level entities
HCM	Healthcare, trauma team training, medical sciences, anesthetists, nurse, patient care, operating rooms, non‐technical skills, cardiac surgery, patient, surgeons, patient‐centered practice, operation, simulation center	Anesthetists, nurse, surgeon, medic, medical emergency teams
EM	Disaster management, information filtering, emergency response, resource management, data aggregation, signal detection, protective actions, warning systems, hazards, organizational citizenship behavior	Emergency managers Manager teams, emergency department, firemen
Training	Training, cognitive skills, simulation, skill development, train simulator study, mental workload, performance, human error, scenario development, adaptation, decision making, simulator	Operators, trainee, learner
Sport	Sport, football, performance player, expertise, sports group, soccer, tactical behavior, team, expertise, game, dynamic‐systems, team cognition, problem detection, decision‐making, expert performance	Players, athletes, coaches, umpires, officials
System autonomy	Automation, agent transparency, adaptive computing systems, robust control, context awareness, social robots, adaptive automation, adaptable automation, artificial intelligence, robotics, cooperation method, consensus, multi‐sensor data, fusion process, coordinated target allocation	Robot, unmanned aerial vehicle, intelligent agent, unmanned aerial vehicle swarm, surveillance robots
NIC	Cybersecurity, networks, information security, cyber, information control, sensor networks, information management, network security, sensor network, information technology security, risk assessment, intelligence service, road safety, communications	Prototype system, information systems, smart applications, sensor, network administrators

Abbreviations: EM, emergency management; HCM, health care and medicine; NIC, network information & communication; T&T, traffic and transportation.

### 2.6 Step 6: Classifying Literature and Adjusting the Fields

Using keywords as the research context and agents as the research object, we classified 3014 studies into their corresponding fields. Six authors conducted a pilot classification of 20 papers and discussed the divergences until they reached a consensus regarding its classification. During the classification process, each paper was classified by two authors, and if there was a divergence, they discussed it with a third author until an agreement was reached. Hence, 173 studies were excluded at this stage because the SA was not applied to any specific field. Moreover, if we found any literature that did not belong to the initial 14 fields, we established a new field and listed the corresponding SA agents and keywords. Hence, three fields, including plant management, building safety, and equipment installation, were proposed.

Steps 5 and 6 were performed iteratively. In the classification process, we further compared the classification criteria for each field to ensure no overlap between the keywords and agent words. After the comparison, we found that in the traffic control and transportation fields, the keywords (traffic, traffic controllers, traffic accident, and transport) and agents (operator and traffic controllers) overlapped. Hence, these were merged into a traffic and transportation field. Similarly, the education and training fields showed an overlap in keywords (training, cognitive skills, simulation, and human error) and agents (trainee and learner). These two fields are merged into a training field. When sorting keywords and agent words for healthcare, we found medical sciences, medical, medical emergency teams, and other words; thus, we expanded the name of the field to healthcare and medicine.

## 3 Results: Major Application Fields of SA

**FIGURE 2:**
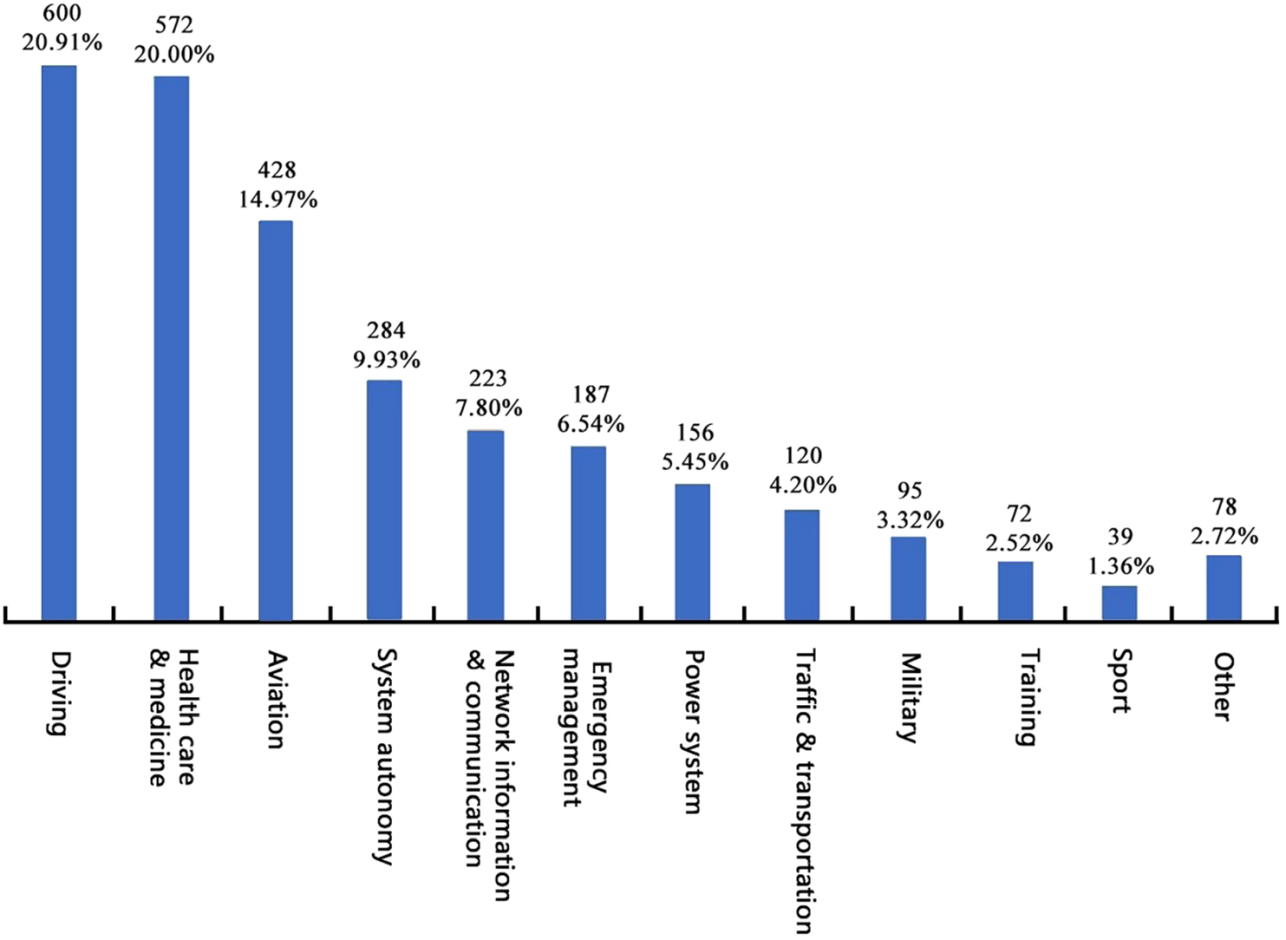
The number and percentage of articles in each field of SA research.

We sorted 11 major fields where SA research was applied, including aviation, driving, power systems, traffic and transportation, healthcare and medicine, emergency management, military, training, sports, system autonomy, and network information and communication (See Table 1 for keywords and agent words in each field). The number and proportion of articles in each field are shown in Figure 2.

The results demonstrated that although SA originated from ergonomic research in aviation, it has expanded to several fields. The most SA studies were found on driving (600 papers). Given the technological advancements in motor vehicles, studies have examined drivers' SA and vehicle interactions in the past three decades. The second most studies were found in healthcare and medicine (572 papers). This result was somewhat unexpected; it reveals the area representing the SA's expansion, as this field is not closely correlated with its point of origin. The third most studies were found in the field of aviation (428 papers). As a pioneering field, SA application in aviation is comprehensive and diverse concerning its utility. Other fields that have produced several studies include systems autonomy, network information and communication, emergency management, power systems, traffic and transportation, military, training, and sports. Additionally, this review categorized fields (oil mining, plant management, construction safety, and equipment installation) with fewer than 20 papers as “others.”

### 3.1 Aviation and Driving

In the fields of aviation and driving, the SA application agents (pilot and driver) were placed in an enclosed cab. Depending on the display interface in the cab, humans could indirectly perceive various types of information and operate the driving machine to perform a task (Liu et al. 2024; Nguyen et al. 2015). In the process of piloting and driving, agents must process various pieces of information related to the cockpit and external driving environment (e.g., abnormal weather and road conditions) to ensure that they do not miss critical information (Nguyen et al. 2015). Compared to novice drivers, more experienced drivers have higher perception efficiency (such as longer dwelling times for crucial information and shorter times for redundant information); meanwhile, they are also more adept at perceiving information feedback from cockpit systems (demonstrated by better attention distribution and more complex and finer visual scanning modes), resulting in a more adequate SA (Gong et al. 2023; Wang et al. 2020). The process of constructing an SA wherein these individuals interact closely with machines to cope with the task environment jointly is a typical characteristic in the fields of aviation and driving.

### 3.2 Power System and Traffic & Transportation

In the fields of power systems and traffic & transportation industries, the agents of SA applications are typically controllers or dispatchers. These individuals are situated in enclosed operating rooms and rely on auxiliary machines or systems (such as supervisory control and data acquisition systems (SCADA), and energy management systems (EMS)) to interact indirectly with the task environment (Miao et al. 2023). Their work is to maintain traffic and power flow and ensure safety (Solberg et al. 2023), requiring them to perform reasonable actions during sudden congestion or complex and diverse management problems (such as before and after a collision or a ship oil spill) within a short period (Wen et al. 2015). These tasks require operators to interact closely with auxiliary machines, as they act as their primary source of information and enable them to perform several operations necessary to complete their task. Therefore, SA is crucial to preserving the security of power and traffic systems (Panteli and Kirschen 2015; Prostejovsky et al. 2019). Insufficient SA may lead to improper coordination between operators and machines in specific emergencies or even routine situations, resulting in operational delays and erroneous actions, ultimately impacting the reliability of the entire power or traffic system (Panteli and Kirschen 2015). There were several instances of large‐scale power outages, which initially began as minor power disturbances, gradually propagated to various regions, ultimately resulting in widespread blackouts because of the operator's failure to respond promptly to alarms issued by the information system (FERC 2011; UCTE 2007). In sum, although power systems and traffic & transportation may differ from aviation and driving, the process of building SA in these fields is remarkably similar, involving close interactions between humans, machines, and the environment.

### 3.3 Healthcare and Medicine

In the healthcare & medical field, SA application agents (i.e., medical practitioners) are predominantly in direct contact with the operating table (Lavoie et al. 2023). Surgical instruments enhance the surgeons' efficiency, accuracy, and safety during surgery, allowing patients to achieve better treatment outcomes and quicker recovery. However, surgical instruments do not provide any contextual information to surgeons, and the operations performed using surgical instruments rely on the surgeon's experience and specific task environment (Yule et al. 2015). Several diagnostic instruments such as stethoscopes, electrocardiographs, and computed tomography (CT) scanners provide real‐time, accurate, and precise medical data that can help medical practitioners quickly understand a patient's exact medical condition, develop better treatment plans, and improve the efficiency and safety of treatment (Boudreault et al. 2023; Cha et al. 2019). Diagnostic instruments perceptually provide effective assistance to medical practitioners; however, medical practitioners must ultimately diagnose the patient's condition and provide effective treatment through direct contact with the patient (direct interaction between human and task environment; Gillespie et al. 2017; Morais et al. 2014). Sufficient SA helps medical practitioners reduce possible human error (Bracq et al. 2021; Schulz et al. 2016). Higher SA is associated with better performance and fewer medical errors during surgery (Yule et al. 2015), anesthesia (Schulz et al. 2016), and emergencies (Khalid et al. 2024). Conversely, poor patient outcomes may be from a lack of SA and failure to immediately recognize or respond to a patient's status (Cha et al. 2019; Flin and Maran 2015).

### 3.4 Military

The interaction pattern in the field of military is close to that of healthcare & medical. The SA application agents primarily involve direct contact and modifying task environments, while machines play a supportive role as tools to enhance human function (Lee et al. 2023). Sufficient SA helps the SA application agents (e.g., soldiers) to reduce possible human error (Bracq et al. 2021; Schulz et al. 2016). For soldiers, the role of rifles is akin to that of surgical instruments for surgeons (Seizovic et al. 2022). Likewise, the role of battlefield reconnaissance equipment like radar, optical telescopes, and thermal imagers is comparable to that of diagnostic instruments employed by medical practitioners (Cave et al. 2020). At high‐risk battlefields, soldiers must quickly and accurately gather critical environmental information before their enemies. The information that battlefield commanders receive from various display devices must be combined with their battlefield experience, tactical understanding, and knowledge of their team's combat capabilities to cope with the battlefield environment (Chapman et al. 2021). They need to collect effective intelligence, analyze the status of both enemy and friendly forces, and take further action (such as reporting to superiors or issuing orders); adequate SA can provide an effective cognitive process for these actions (Lee et al. 2023). An infantry with a superior SA can move safely and effectively, avoid danger in combat, seize critical opportunities to attack the enemy, and quickly transition to a subsequent mission.

### 3.5 Training

In the training field, although it may seem distinct from the above‐mentioned fields of healthcare & medicine and military, SA plays a similar role in reducing human error. Targeted training can help personnel without operational experience quickly accumulate relevant operational experience, enabling them to efficiently establish sufficient SA in similar environments in the future and reduce human error (Loft et al. 2016). The standard method is simulation‐based training, which can provide trainees with an actual, safe, and consistent training environment to effectively improve their SA (Singh et al. 2019). During training, targeted SA scenarios may rapidly and consistently enhance the essential cognitive mechanisms that ordinarily develop through practice experience (Parush 2017; Park et al. 2024). In training situations, the machines that the trainees must learn are a part of the task environment, rather than serving as tools for interacting with the environment. This is explicitly prominent for simulation training, wherein the machine the trainer is working on may not be real but merely a simulator (Korkiakangas et al. 2014).

### 3.6 Sports

The field of sports emphasizes SA distribution and coordination among multiple agents (Weller et al. 2024; Cnossen et al. 2023). SA models in this field include distributed situation awareness (DSA), a theoretical framework that considers multiagency responses from a system perspective. It considers the information held and transacted by humans to complete the tasks in the system under analysis (Stanton, Roberts, et al. 2017; Stanton, Salmon, et al. 2017). Most competitive team sports, such as soccer, basketball, and rugby, increasingly emphasize team strategy. During games, athletes frequently engage in complex team collaborations and construct SA through collaboration. This requires athletes to simultaneously attend to multiple objects in the game environment, including the direction of the ball's movement, the position and direction of teammates and opponents on the field, rapidly changing offensive and defensive situations, fleeting gaps in the field, and engaging in effective team communication (Bourbousson et al. 2015). In team collaboration, athletes constantly maintain awareness of current situational changes and observe team members' behavior as a response to dynamically and continuously coordinate the activities of their teammates (Stanton, Roberts, et al. 2017; Stanton, Salmon, et al. 2017). DSA is the foundation for team dynamic coupling, which can enable athletes to perform better when adequate (Neville et al. 2015). The players of a competitive team can better communicate with the entire team and transact SA, enabling them to maintain and update a higher level of DSA, thus promoting team performance (Neville et al. 2016).

### 3.7 Emergency Management

SA can be applied to emergency management in a manner similar to that in sports. An effective emergency response requires individual emergency management agencies to thoroughly understand the mission context (Keykhaei et al. 2023). For a coordinated response, multiple agencies involved must develop a shared understanding of the affected area and establish a relatively consistent SA, ensuring seamless multi‐agency collaboration (Mohsin et al. 2016). The value of the comprehensive information handled by an emergency decision support system depends on its potential utility for decision‐makers at each SA level, meaning that the higher the SA, the more valuable the information (Fertier et al. 2019). By leveraging the collective intelligence of multiple agencies, emergency managers can better understand “the big picture” during critical situations, and thus make the best, most informed decisions possible for deploying aid, rescue, and recovery operations. Thus, an effective disaster emergency response is based on the successful coordination of multiple human bodies, similar to teamwork in competitive team sports (O'Brien et al. 2020).

### 3.8 System Autonomy

Given the recent advancements in automation and artificial intelligence, autonomous systems (e.g., autonomous robotic vehicles) can reduce or replace human labor under complex circumstances (Kaber 2018). System autonomy aims to develop highly or entirely autonomous systems that can adapt to unpredictable and dynamic situations and complete tasks with less or no human intervention (Endsley 2017). In this field, autonomous systems (machines) closely interact with task environments during SA construction. The system can integrate information about the external environment as perceived by the observation component using data from various internal databases. Subsequently, the system can combine algorithms, integrate data, and orientation components to perform real‐time monitoring of its own system and the external environment, forming SA belonging to the system. Furthermore, the system can make autonomous decisions based on SA with a decision component and then interact with the environment (Ezenyilimba et al. 2023; Gao and Li 2018; Noh 2020). Owing to the considerable challenges in achieving full system autonomy, most systems may operate semi‐autonomously for an extended period (Endsley 2017, 2023). Given this circumstance, both human operators and autonomous systems on human–machine teams form specific SA levels; thus, shared SA between humans and machines must support effective operations (McAree and Chen 2013). However, the existence and development of this field symbolizes that complete system autonomy is possible.

### 3.9 Network Information and Communication

Compared with the emergence of autonomous systems to reduce the operating labor of human beings, machines in the field of network information & communication (NIC) play a more efficient role in processing tasks. Network devices or systems generate and exchange substantial security‐critical and privacy‐sensitive data (Xu et al. 2017). Targeted algorithms and models can improve data processing in information systems to satisfy the demands of specific tasks (such as intelligent transportation, intelligent power, and intelligent medicine) and address security concerns. Anthropomorphic algorithms and models facilitate direct interactions between information systems and task environments. They equip the system with three components: a security situation intelligence detection that can receive massive, multi‐source, and heterogeneous network security information and translate it into comprehensible formats; a situation evaluation that can identify security events, analyze the relationships among these events, and obtain the security situation of the entire network; a situation prediction for predicting the future change trend of the network situation based on the current network security situation, the history of the network situation, and the security situation information (Liu et al. 2024).

## 4 Historical Analysis and Grouping of the SA Fields: Perspectives From HFE

### 4.1 Historical Milestones of Research in the SA Fields

**FIGURE 3:**
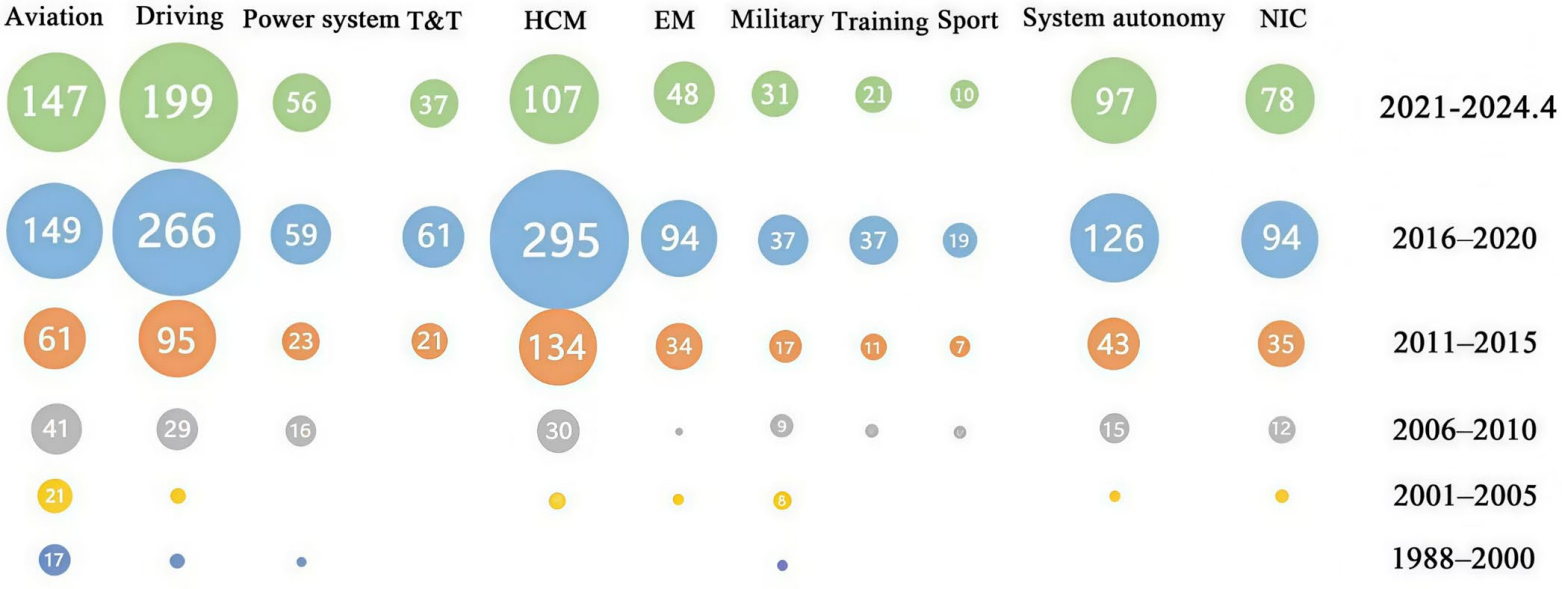
The number of empirical studies published in each field for diverse periods. EM, emergency management; HCM, health care and medicine; NIC, network information and communication; T&T, traffic and transportation.

We traced each field's respective developmental history by depicting their empirical studies published over each five‐year period, and because of fewer studies from 1988 to 2000, we merged these 12 years (Figure 3). We noted that in the early years (1988–2000), there were empirical studies on SA mostly in aviation. Subsequently, during the 15 years from 2006 to 2020, the empirical studies in the field of healthcare and medicine significantly increased, becoming the field with the most empirical literature from 2010 to 2020. Since 2016, the empirical studies in system autonomy have increased quickly and continuously.

### 4.2 Formation and Evolution of SA Groups: An HFE Perspective

From the perspective of HFE (Guastello 2014), we propose that the historical development of these representative fields reflects the fundamental elements of HFE—humans, machines, and task environments, revealing how SA has been evolving by stressing the different aspects of HFE. Originally, SA was developed in studies about the interaction between humans and machines in ergonomic research, such as designing human‐machine interfaces in aviation to facilitate pilots' SA regarding the airplane and the environment (Schnell et al. 2004; Arthur III et al. 2009). Over time, the emphasis on human factors increased, highlighting the importance of understanding how human individuals or teams process information and fulfill tasks even in environments without complicated human‐machine interaction, such as surgical procedures (Yule et al. 2015; Cha et al. 2019). In recent years, with advancements in autonomy and artificial intelligence, the focus has shifted to how machines process information about the task environment and collaborate with humans (Endsley 2023; Ezenyilimba et al. 2023), exemplified by autonomous vehicles. This characterized three groups of SA fields with distinct focuses and historical development, that is, fields center around the aviation field focusing on the ergonomics with human‐machine interaction, fields center around health care and medicine that focus on SA of human‐environment and human‐human interactions, and emerging fields that focus on autonomous machine's SA. Thus, we categorized the SA fields into three groups and named them as the ergonomics‐centered group, the human‐centered group, and the machine‐centered group.

**FIGURE 4:**
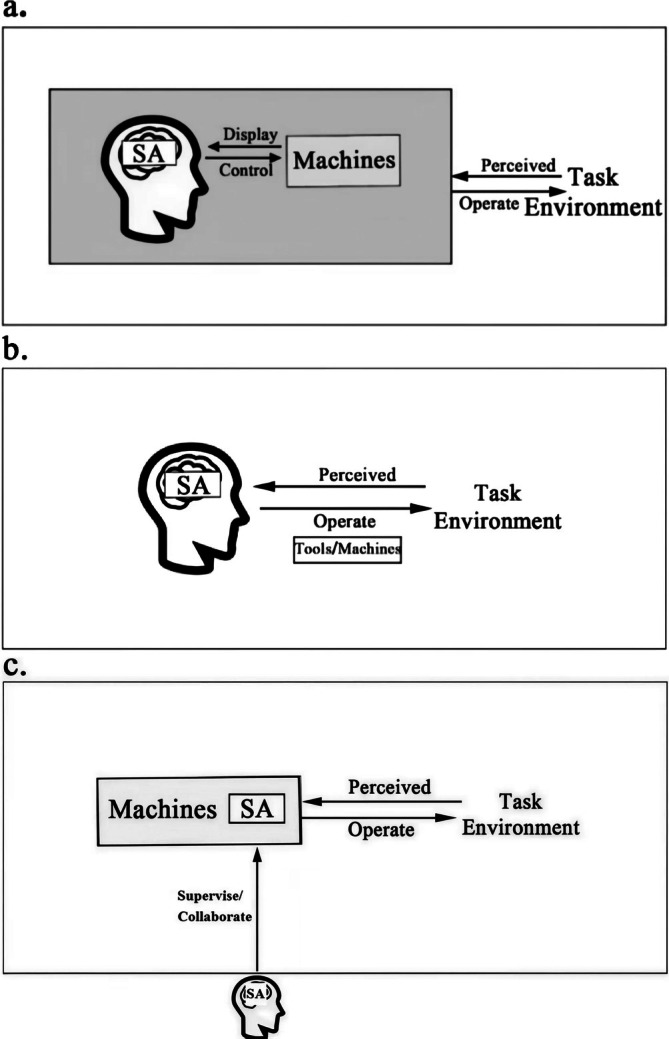
Our framework about SA research from the perspective of HFE. (a) HFE classic setting, human, machine, and environment: Three factors interaction. (b) Human‐centered setting, humans interact directly with the environment. (c) Machine‐centered setting, machines interact directly with the environment.

Regarding historical development, aviation, driving, and power systems are the classic fields of SA origin. These fields share similar approaches concerning application logic, methodology, and models of SA. In these fields, humans and machines interact closely as in classical HFE settings. Specifically, humans and machines interact closely through the human–machine interface with the surrounding task environment, and SA emerges from the interaction between the operator and machine. Human operators obtain information on environmental situations primarily from interacting with the machine, thereby forming an SA, and then operate in the environment indirectly through the machines (see Figure 4a). Notably, although the empirical literature on SA in traffic and transportation came later, it also involves a close interaction between operators and machines. Accordingly, this review classifies these fields (aviation, driving, power systems, and traffic and transportation) that stress the SA from human–machine interaction in classical HFE settings as the ergonomics‐centered group (see Table S1).

Since 2006, the rapid SA development in the healthcare and medical field has benefited from numerous innovative SA theories and models. For example, in 2003, Fletcher considered SA to be a part of the anesthetists' non‐technical skills system. This field seems not closely related to the initial SA fields intertwined with ergonomics (e.g., aviation), and the HFE settings have also changed (Bourbousson et al. 2015; Yu and Han 2019). This variant of the HFE focuses more on the interactions between humans and the environment (see Figure 4b). In this setting, the SA application agents remain humans; however, they contact the environment directly or through the operation of tools rather than indirectly contacting the environment through machines. This interactive feature is also found in military, emergency management, training, and sports fields. This review clustered the five fields that conform to this setting and named them the human‐centered group (see Table S2).

The empirical literature in the areas of system autonomy and NIC has continually increased since 2016 owing to developed computational intelligence and learning algorithms. Automation and system autonomy technologies enable machine systems to perform tasks with limited or no human intervention (Endsley 2017). Currently, machines that interact closely with humans in traditional HFE settings are becoming increasingly autonomous and interacting independently with the environment. This variant of HFE focuses on the interaction between the machine and the environment (Figure 4c). SA agents have been transformed from humans into machines. The machines collect data from the environment, generate an SA belonging to the machine systems, and then interact with the environment (van de Merwe et al. 2024). In the interaction process, machines hold a great portion of SA, while humans involved in the process also hold certain SA, assuming a supervisory or collaborative role. The rise of machines encompasses two different application purposes: to help reduce repetitive labor for humans and handle tasks that humans cannot perform. System autonomy and NIC primarily correspond to these two purposes (Endsley 2017; Liu et al. 2020). This review clustered the two fields into a machine‐centered group (see Table S3).

### 4.3 Quantitative Analysis of SA Group Evolution

Before 2000, there were a small number of empirical studies on SA, mostly in the fields comprising the ergonomics‐centered group but only a few in the human‐centered group and none in the machine‐centered groups. Subsequently, there has been rapid growth in SA research within the ergonomics‐centered group and even more rapid growth in the human‐ and machine‐centered groups. From 2001 to 2010, 201 empirical studies were conducted across the three groups: 111 in the ergonomics‐centered group (55.22% of the total), 58 in the human‐centered group (28.85%), and 32 in the machine‐centered group (15.92%). From 2011 to 2020, 735 were in the ergonomics‐centered group (42.91%), 680 were in the human‐centered group (39.70%), and 298 were in the machine‐centered group (17.35%). We calculated the growth ratio of SA empirical studies across the three groups over a 10‐year period. Between 2011 and 2020, the growth ratios were 562% in the ergonomics‐centered group (from 111 to 735), 1072% in the human‐centered group (from 58 to 680), and 1088% in the machine‐centered group (from 32 to 380).

This review highlights that the evolution of the three SA groups reflects the 40 years of historical development of the SA concept and its gradual application across various fields. SA was initially introduced in the aviation field, where the ergonomics‐centered group focused on interactions between humans, machines, and the task environment. Over time, this expanded into the human‐centered group, which emphasizes the interaction between humans and the task environment. Lately, the focus shifted to the machine‐centered group, which prioritizes the interaction between machines and the task environment.

**FIGURE 5:**
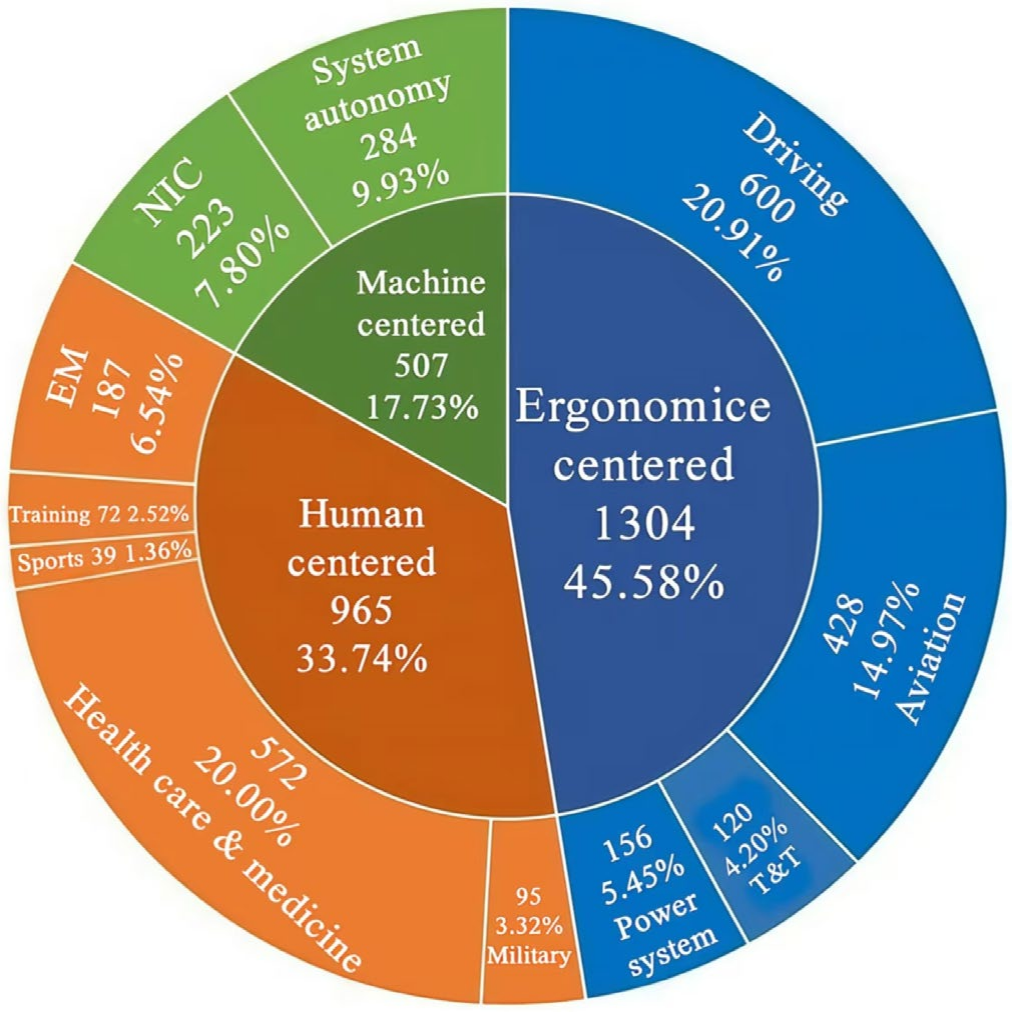
The categorization of SA fields into the ergonomics‐, human‐, and machine‐centered groups. EM, emergency management; NIC, network information and communication; T&T, traffic and transportation.

In summary, we categorized the 11 major fields that produced SA studies into three groups (see Figure 5): ergonomic‐centered, human‐centered, and machine‐centered. This categorization is based on the classic HFE framework and its two variants, which emphasize the diverse roles played by humans and machines in interacting with task environments. This clustering method can help understand the focus of the extension of the SA concept to various fields and the diverse characteristics of its applications in these fields.

### 4.4 Recent Development of the SA Groups

#### 4.4.1 Recent Development of the Ergonomics‐Centered Group

In recent years, SA applications in the four fields of ergonomic‐centered groups have been undergoing significant changes. In aviation and driving fields, highly automated driving (HAD) technology has been a significant breakthrough that reduced the workload and improved safety, comfort, and efficiency for drivers and pilots (Röckel and Hecht 2023). Several types of driving behaviors originally operated by humans are gradually being represented by machines (Ignatious et al. 2023). Drivers can release their hands from the steering wheel and are no longer required to permanently monitor the road. In this driving scenario, the HAD system can perform the primary driving tasks, allowing drivers to engage in nondriving activities, such as using mobile phones, reading books, or performing office work (de Winter et al. 2014; Zangi et al. 2022). The HAD technology is profoundly transforming the conventional practice of relying solely on human drivers to operate vehicles, thereby transferring the driving task to the automated driving system.

Similarly, driven by artificial intelligence (AI) and leveraging the Internet of Things (IoT) and 5G communication technology, several intelligent settings, digital interfaces, and digital systems have been developed in the fields of power, traffic, and transportation (Ezenyilimba et al. 2023; Noh 2020). These advancements have reduced the repetition of meaningless operations and operational risks and increased operational efficiency (Endsley 2023; Camblor et al. 2024). Among these, smart grids are representative, which are a paradigm introduced into conventional electricity networks to enhance how generation, transmission, and distribution networks interrelate (Esenogho et al. 2022). Through large‐scale data analysis and predictive technology, intelligent systems can automatically achieve the dynamic optimization of power systems, thereby improving the stability, reliability, and economy of the power grid. Additionally, by combining IoT technology, a smart grid system can dynamically adjust to user demands and provide more personalized power services to meet varied user needs (Shafique et al. 2020).

#### 4.4.2 Recent Development of the Human‐Centered Group

The SA application in various fields of the human‐centered groups are also undergoing drastic changes. The development of intelligent technology has significantly improved the practicability of various types of equipment (Bisht et al. 2018). Advanced equipment has become crucial to medical and military operations (Anya and Tawfik 2017; Seizovic et al. 2022). For instance, visualization technologies have enhanced clinicians' SA by helping them effectively perceive and verbalize underlying medical issues, improving diagnostic confidence, and reducing workload (Gasciauskaite et al. 2023; Tscholl et al. 2019). Sensory enhancement equipment can provide soldiers with more battlefield environment data and assist them to make effective battlefield judgment to help soldiers form adequate SA (Clasing and Casali 2014).

With improving smart city systems and the rise of social media, several disaster‐related data, texts, and photos have become available online after or when an emergency occurs (Shan et al. 2019), which may contain some high‐value available information (Gao et al. 2018). In these situations, manual integration may be inefficient. Several decision systems for various disasters have been gradually developed based on big data and machine learning, allowing reduced human labor and minimizing human errors (Zhong et al. 2022).

#### 4.4.3 Recent Development of the Machine‐Centered Group

Higher autonomy levels are being developed to integrate information across disparate data repositories, create real‐time health monitoring, and for several other applications (Endsley 2017). As the algorithms and models of autonomous systems continue to improve and update, they will better understand and process data in task environments, allowing them to adapt to extended new task environments and make more precise predictions and operations (Danial et al. 2019; Wei et al. 2020). Studies aim to make autonomous systems more transparent for users and stakeholders to better understand the foundation and fundamentals of their decisions (Vered et al. 2020). This can enhance the credibility and admissibility of the intelligent autonomous systems. Concerning cybersecurity, studies are exploring various new technologies and strategies such as multifactor authentication, encryption, and blockchain to ensure the security and reliability of autonomous systems for processing and storing extended sensitive data (Zhou et al. 2022).

Furthermore, machines and autonomous systems are increasingly collaborating with humans, forming human‐machine systems. While a machine typically functions as an individual actor, a system focuses on the collective cognitive processes within a joint cognitive system (Artman and Garbis 1998). In terms of SA possession, systems and traditional machines operate at different levels. While SA can be viewed as the possession of an individual actor (whether human or machine), in the context of systems, the system itself becomes the unit of analysis. In a system, SA comprises a network of information upon which different components of that system have distinct views and ownership. The systems' SA is distributed across team members, partly shared and partly unique to the agents involved, whether they are machines or humans (Artman and Garbis 1998; Stanton, Roberts, et al. 2017; Stanton, Salmon, et al. 2017). The key to distributed SA in the human‐machine systems is ensuring that different perspectives are interconnected, so that the appropriate information is given to the right member at the right time. Essentially, while traditional machines emphasize individual‐level SA, systems highlight the dynamic interaction and shared cognition between machines and humans (Stanton, Roberts, et al. 2017; Stanton, Salmon, et al. 2017).

In human‐machine systems, human‐autonomy teaming (HAT) is critical (Endsley 2023; Johnson and Vera 2019). Shared SA between team members is essential for managing interdependencies and aligning goals (Endsley 2023; National Academies of Sciences, Engineering, and Medicine 2021). It is not only important for both the autonomous system and the human to have accurate SA for their respective tasks, but also for both to share relevant information. Moreover, humans need to understand the capabilities and limitations of autonomous systems, as well as to have real‐time clarity on the autonomous system's SA within the current context (National Academies of Sciences, Engineering, and Medicine 2021; O'Neill et al. 2022). This understanding enables human teammates to adjust their behavior accordingly, and determine when the system can be trusted and when tasks should be performed manually. Achieving this requires the explainability and transparency of autonomous systems, which have become increasingly critical areas of research (Endsley 2023). Explainability refers to describing the logic, processes, factors, or reasoning behind the system's actions or recommendations, while transparency emphasizes making the autonomous system's behavior understandable and predictable (Endsley 2023). Explainability provides retrospective insight into the system's processes, revealing why the system takes certain actions (system capabilities or processes), helping users build accurate mental models of autonomous systems, thereby indirectly supporting SA. Transparency, particularly display transparency, focuses on what is happening, offering real‐time support for SA by providing current and prospective situational information through the system's interface. Building upon explainability and transparency, recent SA research has developed frameworks to enhance human perception, comprehension, and projection in HAT. The Situation Awareness Framework for Explainable AI (Sanneman and Shah 2022) and the SA‐based Agent Transparency Model (Chen et al. 2018) are examples of such frameworks.

To assess and reveal autonomous systems' SA, it is necessary to develop appropriate metrics and techniques. The methods for measuring SA may vary depending on the type of agent, as different agents—such as machine learning‐based agents and logic‐based agents—may exhibit distinct characteristics of SA (Ganesh et al. 2022; Kridalukmana et al. 2022). Machine learning‐based agents can automatically identify complex associations between sensor data and situations using machine learning algorithms, but they do not have explicit, encoded rules, making it challenging for humans to comprehend their SA and how it is achieved. Logic‐based agents operate according to predefined functions and logical rules, relying on expert knowledge representations and reasoning techniques to infer situations from data, which constrains their effectiveness to environments with a limited number of sensors and relationships among events (D'Aniello and Gaeta 2023). Currently, metrics and techniques for assessing autonomous systems' SA are being actively explored. One proposed method involves measuring “surprise,” defined as a stimulus that deviates from an agent's expectations (Dahn et al. 2018). For agents that predict future states, surprise can be quantified as the difference between predicted and actual situations. This can be assessed in real time, even by the agent itself. Agents can recognize its lack of SA based on surprise and take actions to regain it, such as acquiring additional information. They can also use surprise as a cue to improve their internal models and routines. In addition to surprise, more sophisticated metrics, such as “situation consciousness,” are being developed to assess the quality of an agent's SA. This metric incorporates factors like context quality, degree of consistency, and coverage quality, providing a comprehensive description of the agent's SA state (Müller et al. 2024). To effectively communicate and visualize agents' SA states, intelligent Digital Twins (iDTs) can be employed (Pairet et al. 2019). Digital Twin is a “virtual representation of a physical asset” (Ashtari Talkhestani et al. 2018), while iDTs further incorporates aspects of intelligence such as data analysis and reasoning. By utilizing sensors and visualization devices, iDTs enable simulations and real‐time communication with human operators, offering high‐level indicators of agents' comprehension of the current situation (D'Aniello and Gaeta 2023). These metrics and techniques are particularly effective for assessing and communicating the SA of machine learning‐based agents, while logic‐based agents may require modified or additional methods. As research progresses, these two agent types will likely converge, and the hybrid approaches, combining the advantages of learning‐based and knowledge‐based approaches, represent a promising research direction for addressing challenges in SA in complex systems (D'Aniello and Gaeta 2023).

### 4.5 Future Research Directions for SA Groups

Our classification of the three groups provides an overall comprehensive understanding of SA literature, highlighting the theoretical and methodological emphasis of each group. This classification can help understand the characteristics of numerous SA studies in various fields, demonstrating how fields within each group are similar to each other and how the research differs between groups. Furthermore, we provide a quantitative analysis of the growth in the number of publications across the three groups from 2021 to April 2024, and thereby forecast the future development trends of each group, highlighting the exchange and integration among them.

**FIGURE 6:**
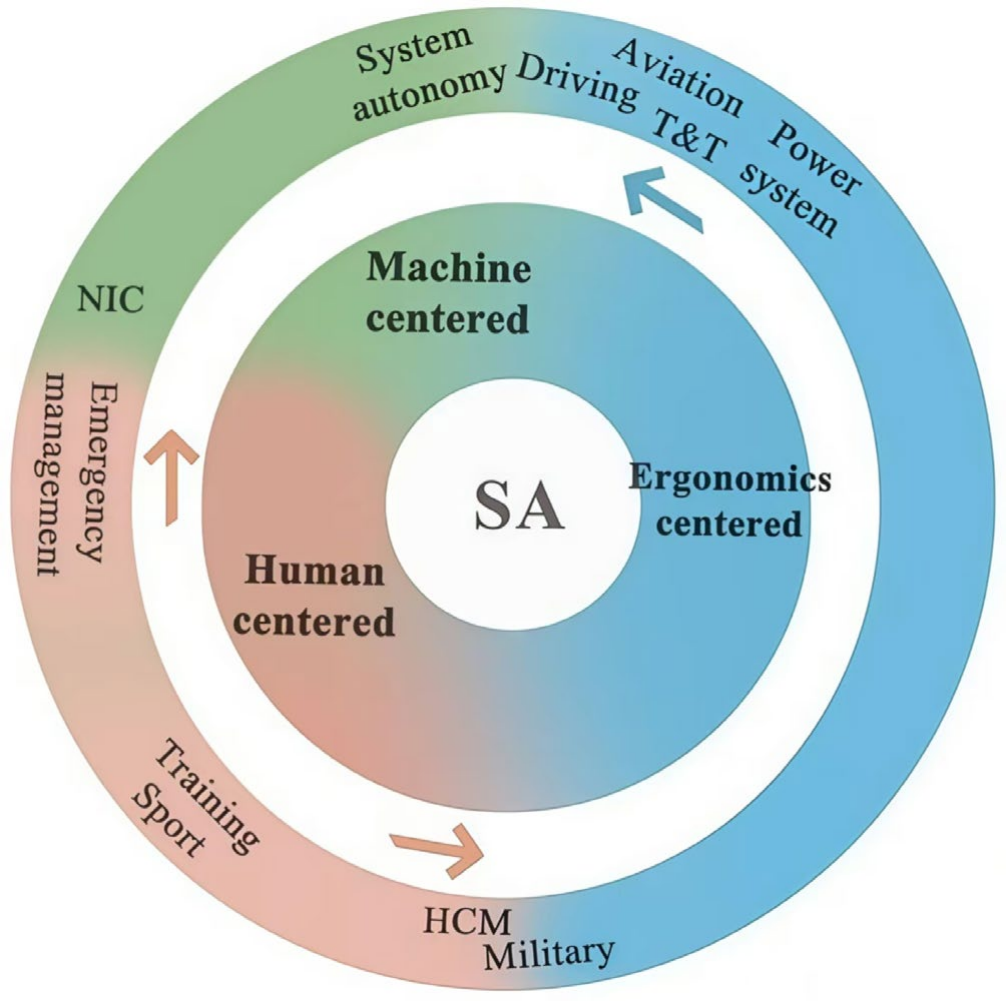
The developing trend of SA application fields in the three groups. HCM, healthcare and medicine; NIC, network information and communication; T&T, Traffic and transportation.

In the future, the SA application is likely to continue to evolve across these three groups. The ergonomics‐centered group may remain a primary SA research focus. Among the 831 newly published empirical studies on SA from 2021 to April 2024, 439 are from the ergonomics‐centered group, making it the largest among the three groups and accounting for 52.83% of the total. The emergence of HAD demonstrates the possibility of autonomous driving using autonomous systems. With technology development, the aviation and driving fields may gradually converge to machine‐centered groups. The research direction is increasingly focusing on the construction and optimization of autonomous systems' SA, the patterns of change in human operators' SA under autonomous driving modes, and how human operators can take over vehicle control based on their own experience to ensure driving safety when the autonomous driving system suddenly fails (Linkov and Vanžura 2021; Park 2019). Similarly, in power systems, and traffic and transportation fields, with the rise of machine operation space, the initial interaction situation of humans, machines, and the environment is gradually biased toward the interaction between machines and the environment. Therefore, the fields in the ergonomics‐centered group are gradually shifting to the machine‐centered group in a way close to system autonomy (Figure 6).

On the other hand, the human‐centered group may experience further expansion. Among the recent literatures on SA from 2021 to 2024, the human‐centered group accounted for 217 out of 831 studies (26.11%), related to the personnel performance in a wide and continuously expanding range of fields concerning human activity. With emerging advanced technologies, tools that were originally solely auxiliary are becoming intelligent to work with humans. During SA construction in the fields of healthcare and medicine and military, the auxiliary role of intelligent equipment is gradually changing, and medical practitioners and soldiers are increasingly interacting with them during their missions. Therefore, these two fields may gradually converge towards the ergonomics‐centered group. Furthermore, technology has made machines increasingly critical in execution. For instance, various disaster decision‐making systems based on big data and machine learning are establishing machines as key agents of execution, transitioning the interaction structure from human‐environment to machine‐environment (Zhong et al. 2022). As a result, emergency management is gradually shifting from the human‐centered to the machine‐centered group in a way similar to NIC (Figure 6).

Meanwhile, literature in the fields of system autonomy and NIC reached 175, accounting for 21.06% of the total, indicating that the machine‐centered group will continue growing as AI and other technology develop. Autonomous systems are likely to continuously develop and advance to become intelligent, transparent, and secure. NIC will increasingly exhibit the characteristics of automated defense systems. Machine systems will be capable of independently handling various tasks. Fields wherein machine systems play a pivotal role may continue to increase. The two substantial application methods of autonomous systems, including reducing repetitive human labor and processing tasks that humans cannot complete, may be further strengthened. Increasingly, more fields requiring these two aspects may integrate in machine‐centered groups.

In sum, alongside the respective development of the three groups, they are also gradually converging. Future studies can exploit the respective advantages of SA in humans and autonomous systems, improving them complementarily and enabling them to interact dexterously in various complex‐task environments.

### 4.6 Limitations

Due to the nature of our review, selection, and filtering processes, this review included only journal articles, excluding conference papers for greater rigor. Additionally, we excluded unpublished and non‐English language articles, which may introduce publication and language biases. Future studies should address these limitations.

## 5 Conclusion

This study conducted a systematic review of SA literature, including 2860 empirical studies between 1975 and 2024. We divided empirical studies applied to SA into 11 major fields (including aviation, driving, power systems, traffic and transportation, health care and medicine, emergency management, military, training, sport, system autonomy, and network information and communication), indicating that the SA application has substantial diversity and interest in each field is high. By analyzing the associations and distinctions between fields, this review further clusters the 11 fields into three groups (ergonomic‐centered, human‐centered, and machine‐centered) according to their different interaction focuses on humans, machines, and task environments. Thus, our findings provide a feasible classification of SA application fields, as well as insights for future SA research. In the future, as a key component of human and machine information processing, SA's application value may be recognized in more fields; thus, SA research in the three groups may continually expand. Various fields applying SA may learn from and integrate with each other to derive more unified theoretical models and efficient methods to deal with various forms of interactions among humans, machines, and task environments.

## Conflicts of Interest

The authors declare no conflicts of interest.

## Supporting information


**Data S1.** Supporting Information.
