# Supplementary material for: A Systematic Review of Empirical Studies on Situation Awareness: Perspectives From the Interaction Among Humans, Machines, and the Task Environment
Source: Psych J. 2025 Jul 6;14(5):718–33. doi: 10.1002/pchj.70027 (PMC12520847; doi:10.1002/pchj.70027)
Supplement: Supplementary file 1 — Data S1. Supporting Information. [file PCHJ-14-718-s001.docx]

**Supplementary Table 1** The Parameters of Ergonomics-Centered Group

| Field | Agents (Human) | Machine/tool | Task Environment |
| --- | --- | --- | --- |
| Aviation | pilots, runway controllers, tower controllers | cockpit, flight decks,air traffic control system, unmanned aircraft system, autopilot system | flight environment, weather environment, air traffic environment, equipment management environment |
| Driving | drivers, driver assistance systems | dashboard, driver assistance systems, highly automated driving system | road environment, traffic environment, weather environment, vehicle environment |
| Power system | operator, operations team | supervisory control and data acquisition system, power monitoring system, power load management system, programmable logic controller | power supply environment, power demand environment, equipment reliability environment, safety monitoring environment |
| T&T^1^ | operator, traffic controllers, crew, accident signaling system, control centers | control system, accident signaling system, road monitoring system, traffic flow detector, vehicle tracking system | road traffic environment, weather environment, traffic accident environment, traffic signal management environment |

**Supplementary Table 2** The Parameters of Human-Centered Group

| Field | Agents (Human) | Machine/tool | Task Environment |
| --- | --- | --- | --- |
| HCM^2^ | anesthetists, nurse, surgeon, medic, medical emergency teams | Surgical instruments, syringe, electrocardiogram machine, X-ray machine, CT scanner | assessment and diagnosis environment, treatment environment, equipment management environment, patient communication environment |
| Military | cadets,warfighters, soldiers, infantry squads, incident commanders, higher command level entities | weapons, tactical radars, communication equipment, navigation equipment | combat environment, tactical environment, intelligence environment, terrain and weather environment |
| EM^3^ | emergency managers, manager teams, emergency department, firemen | emergency response equipment, first aid equipment, life support systems communication equipment, search and rescue equipment | emergency response environment, disaster relief environment, critical incident management environment |
| Sport | players, athletes, coaches, umpire, officials | training equipment, activity trackers, communication equipment | game environment, team communication environment, technology environment |
| Training | operators, trainee, learner | training simulator, teaching aid, instructional video | training environment, equipment management environment |

**Supplementary Table 3** The Parameters of Machine-Centered Group

| Field | Human | | Machine/tool | Task Environment |
| --- | --- | --- | --- | --- |
| System autonomy | supervisor | autonomous system, robot, unmanned aerial vehicle, intelligent agent, unmanned aerial vehicle swarm, surveillance robots | | autonomous decision-making environment, adaptive learning environment, data management environment, self-optimization environment |
| NIC^4^ | supervisor | router system, web server, proxy server, information storage, firewall, load balancer, intrusion prevention system | | network security environment, network monitoring environment, network management environment, network analysis environment |

# Note

1. T&T, Traffic and transportation

2. HCM, health care and medicine

3. EM, emergency management

4. NIC, network information & communication
